# Association between Triglyceride-Glucose Index and Type 2 Diabetes Mellitus in the Japanese Population: A Secondary Analysis of a Retrospective Cohort Study

**DOI:** 10.1155/2020/2947067

**Published:** 2020-12-11

**Authors:** En-qian Liu, Ya-ping Weng, Ai-ming Zhou, Chun-lai Zeng

**Affiliations:** ^1^Department of Cardiology, Lishui Hospital, Zhejiang University School of Medicine, Lishui, 323000 Zhejiang, China; ^2^Department of Gastroenterology, Lishui Hospital, Zhejiang University School of Medicine, Lishui, 323000 Zhejiang, China; ^3^Department of Critical Medicine, The First Affiliated Hospital of Wenzhou Medical University, Wenzhou, 325000 Zhejiang, China

## Abstract

Triglyceride-glucose index (TyG index) is associated with type 2 diabetes mellitus (T2DM), but research on this relationship is limited in Japan. The purpose of this study was to evaluate the correlation between TyG index and the risk of T2DM in the Japanese population. Here, 12732 participants were selected from the NAGALA study (NAfld in the Gifu Area, Longitudinal Analysis) conducted between 2004 and 2015 for a retrospective cohort analysis. The association between TyG index and T2DM was assessed using the Cox proportional-hazard model. Subgroup analyses were conducted according to age, sex, smoking status, alcohol consumption, waist circumference, BMI, and follow-up duration. The formula for TyG index was expressed as ln [fasting triglyceride level (mg/dL) × fasting plasma glucose level (mg/dL)/2]. After follow-up, 150 (1.18%) patients developed T2DM. After adjusting for potential confounders, a linear relationship was observed between TyG and the risk of T2DM. After adjusting for age, sex, BMI, waist circumference, HDL-cholesterol, total cholesterol, systolic blood pressure, regular exercise, smoking status, and alcohol consumption, TyG index, as a continuous variable, was associated with an increased risk of T2DM (adjusted hazard ratio (aHR), 1.79; 95% confidence interval (95% CI), 1.25-2.57). Compared with the first quartile of TyG index, subjects in the fourth quartile were 2.33-fold more likely to develop T2DM (aHR 2.33, 95% CI 1.09-4.96; *P* for trend 0.0224). Subgroup analyses showed that the association between TyG index and incident T2DM stably existed in different subgroups according to the variables tested. Therefore, TyG index was linearly related to the risk of incident T2DM in the Japanese population and may be used as a monitoring tool.

## 1. Introduction

At present, epidemiological reports suggest 463 million people have diabetes worldwide, and this number is projected to increase to 700 million people by 2045 [1]. Type 2 diabetes mellitus (T2DM) and its associated complications impose an enormous economic burden on individuals, societies, and nations. Major risk factors for T2DM include an unhealthy diet, obesity, and a sedentary lifestyle [2, 3]. Early and effective identification of diabetes can decrease early morbidity and improve patient quality of life. Large epidemiological investigations require noninvasive and inexpensive tests to identify those with a greater risk of developing T2DM and to prevent its development.

To our knowledge, the key pathogenesis of T2DM involves islet *β*-cell dysfunction and insulin resistance (IR) [3, 4]. IR manifests such that insulin-dependent tissues (e.g., skeletal muscle, fat, and liver) do not respond appropriately to insulin stimulation [5]. IR is a feature of various metabolic disorders such as hyperglycemia and hypertriglyceridemia [5]. Simental-Mendía et al. [6] proposed that triglyceride-glucose (TyG) index, which is estimated using the formula ln (fasting triglycerides (mg/dl) × fasting blood glucose (mg/dl)/2), is associated with diabetes. In fact, TyG index showed a remarkable relationship with commonly used surrogate indicators of IR, such as hyperglycemic clamp and the homeostasis model assessment of IR (HOMA-IR) [6–9].

As the TyG index is a reliable and alternate indicator of IR, it may be used to assess the risk of diabetes. It can be easily obtained and calculated in clinical practice or large-scale epidemiologic investigations. Certainly, several studies have suggested that TyG index is significantly associated with the risk of developing T2DM in China [10, 11], Singapore [12], Europe [13, 14], Korea [15, 16], Thailand [17], and Iran [18, 19]. However, the relationship between TyG index and T2DM has not yet been studied in Japan. Here, study data were downloaded freely and a secondary analysis was performed [20]. Our research goal was to evaluate the correlation of TyG index with the risk of developing incident T2DM.

## 2. Methods

### 2.1. Data Source

The DATADRYAD database (http://www.Datadryad.org/) allows researchers to freely download the original study data. The data were cited from the dryad data package (Okamura, Takuro et al. (2019)). Data can be obtained from the following: Ectopic fat obesity presents the greatest risk for incident type 2 diabetes: a population-based longitudinal study (dataset: 10.5061/dryad.8q0p192). The database materials included the following variables: age, sex, waist circumference (WC), weight, body mass index (BMI), systolic blood pressure (SBP), diastolic blood pressure (DBP), alanine aminotransferase (ALT), fasting plasma glucose (FPG), total cholesterol (TC), aspartate transaminase (AST), *γ*-glutamyltranspeptidase (GGT), high-density lipoprotein cholesterol (HDL-C), smoking status, exercise, fatty liver, alcohol consumption, triglyceride (TG), hemoglobin A1c (HbA1c), obesity phenotype, obesity, visceral fat obesity, ethanol consumption, diabetes mellitus (DM), and follow-up duration.

### 2.2. Study Population

In the Murakami Memorial Hospital (Gifu, Japan), there was a medical examination project aimed at finding chronic diseases and improving public health. Okamura et al. [20] reported that obesity phenotypes have an impact on the risk of developing T2DM by analyzing the NAGALA (NAfld in the Gifu Area, Longitudinal Analysis) database. Participants received an examination between 2004 and 2015, and 60% of the participants had one to two exams per year. A total of 12723 participants (6175 men and 6548 women) were selected according to the following exclusion criteria: (1) the participants had missing related data; (2) the participants had hepatitis B or C virus or fatty liver disease; (3) the participants had a heavy drinking habit (alcohol consumption over 60 g/day for men and 40 g/day for women); (4) the participants took medication at baseline; (5) the participants' fasting plasma glucose was greater than 6.1 mmol/L at baseline, which did not meet the underlying assumption of proportional hazards. The ethics committee of Murakami Memorial Hospital authorized the study, and each participant provided written informed consent.

### 2.3. Data Collection and Measurements

The NAGALA database contains the clinical history and lifestyle factors of participants based on a standardized questionnaire. For example, alcohol consumption was recorded by the type of alcohol and mean weekly alcohol volume of the past month and then divided into four groups: no or minimal drinker (<40 g/week), light drinker (40–140 g/week), moderate drinker (140–280 g/week), or heavy drinker (>280 g/week) [21]. Likewise, for smoking status, participants were classified as never, ex, or current smoker. Exercise status was characterized as regular if any sport that lasted long enough to produce perspiration > 1 × /week was performed, such as jogging, bicycling, and swimming [22]. Fatty liver was examined by abdominal ultrasonography and diagnosed by gastroenterologists. Of the four criteria (hepatorenal echo contrast, liver brightness, deep attenuation, and vascular blurring), the subjects who had liver brightness and hepatorenal contrast were diagnosed with fatty liver disease [23]. The formula of TyG index was as follows: ln [fasting triglyceride level (mg/dL) × fasting plasma glucose (FPG) (mg/dL)/2] [6]. Finally, T2DM was defined as any of the following: fasting plasma glucose ≥ 7 mmol/L, HbA1c ≥ 6.5%, or self-reported [20].

### 2.4. Statistical Analysis

Continuous variables (normal distribution) are presented as mean with standard deviation (SD) and continuous variables (skewed distribution) are expressed as median with interquartile range (IQR). Categorical variables are presented as frequencies and percentages. We used the chi-square test, one-way analysis of variance, or Kruskal-Wallis test to examine the statistical differences in the groups stratified by TyG index quartiles. We employed the univariate and multivariate Cox proportional hazard models to assess the relationship between TyG index and the risk of T2DM. We used three models: model 1, a crude (univariate) model; model 2, adjusted for age and sex; and model 3, adjusted for age, BMI, sex, waist circumference, regular exercise, HDL-C, TC, alcohol consumption, smoking status, and SBP. In the models, we used a median value in each quartile of TyG index to perform the linear trend tests. In addition, the nonlinear relationship between TyG and T2DM was estimated using the Cox proportional hazards regression model with cubic spline functions. To find modifications and interactions, we used a stratified linear regression model and likelihood ratio test in the different subgroups according to sex, age (<45 years or ≥45 years), smoking status (never, past, or current), alcohol consumption (0 g/week or >0 g/week), waist circumference (<90 in men, <80 in women or ≥90 in males, ≥80 in females), BMI (<25 kg/cm^2^ or ≥25 kg/cm^2^), and follow-up duration (<1982 days or ≥1982 days, according to median of follow-up duration). We used the Statistical Packages R (The R Foundation, Vienna, Austria) to analyze the data. When the calculated *P* value was less than 0.05, the statistical difference was considered significant.

## 3. Results

### 3.1. Baseline Characteristics of Selected Participants

A total of 12723 participants were assessed in this retrospective cohort study.  Table 1 displays the baseline characteristics of the participants grouped by quartile of TyG index. Overall, the mean age of the 12723 participants was 43.47 ± 9.01 years, and 48.53% were male. After follow-up, 150 participants developed T2DM with an incidence of 1.18%. Participants in the highest group of TyG (Q4) had higher values of age, BMI, WC, TC, TG, FPG, SBP, and DBP and consisted of more males, smokers, and drinkers than the other groups (Q1-3). Participants with the highest TyG (Q4) had lower values in HDL than those with lower TyG (Q1-Q3).

### 3.2. Univariate Analysis for T2DM

Table 2 lists the results of the univariate analysis for the association between risk factors and T2DM. Using the univariate Cox proportional hazard model, we discovered that regular exercise, light alcohol consumption, and past smoking were not associated with T2DM. We also found that T2DM was associated with lower HDL-C. In contrast, univariate analysis showed that males, age, BMI, WC, TC, TG, FPG, SBP, DBP, moderate and heavy alcohol consumption, current smoking, and TyG index were positively associated with T2DM.

### 3.3. Unadjusted and Adjusted Cox Proportional Hazard Model

We used Cox proportional hazard models to assess the independent effects of TyG index on the risk of developing T2DM (univariate and multivariate Cox proportional hazard models).  Table 3 lists the effect sizes (hazard ratio (HR) and 95% confidence intervals (95% CI)). In the unadjusted model (model 1), an increase in TyG index of one unit was associated with a 2.94-fold higher risk of incident T2DM (HR 2.94, 95% CI 2.28-3.80). In model 2, an increase in TyG index of one unit increased the risk of developing T2DM by 2.26-fold (HR 2.26, 95% CI 1.70-3.01) after adjusting for age and sex. In model 3, each additional unit of TyG index was associated with a 1.79-fold higher risk of incident T2DM (HR 1.79, 95% CI 1.25-2.57). For sensitivity analysis, TyG index was transformed into a categorical variable (quartile of TyG index), and the *P* value for the trend of TyG index with categorical variables was consistent with the result of TyG index as a continuous variable in the different models.

### 3.4. Threshold Effect Analysis of TyG on Incident T2DM

**Figure 1:**
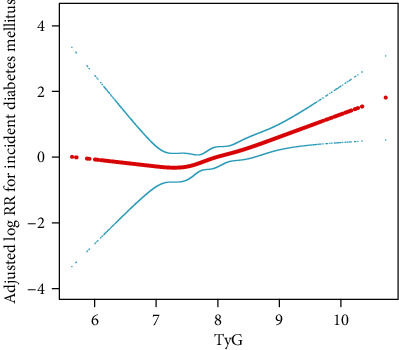
Dose-dependent relationship between TyG and incident diabetes mellitus. Solid red line represents the smooth curve fit between variables. Blue bands represent the 95% confidence interval of the fit. Data were adjusted for age, sex, BMI, WC, HDL-C, TC, SBP, regular exercise, smoking status, and alcohol consumption (continuous).

We used a Cox proportional hazards regression model with cubic spline functions to evaluate the relationship between TyG index and incident T2DM (Figure 1). After adjusting for age, BMI, sex, WC, regular exercise, HDL-C, TC, alcohol consumption, smoking status, and SBP, TyG index and T2DM had a positive linear relationship.

### 3.5. Subgroup Analyses

Subgroup analyses for the correlation between TyG index and incident T2DM are presented in Table 4. The participants were divided into subgroups according to age, sex, smoking status, alcohol consumption, WC, BMI, and follow-up duration. The results showed that the association between TyG and incident T2DM stably existed in the different subgroups.

## 4. Discussion

In our population-based retrospective cohort study, we observed that TyG index, as a continuous or categorical variable, was positively, linearly related to T2DM after controlling for covariates in the Japanese population. The results were stable in subgroups according to sex, age, smoking status, alcohol consumption, WC, BMI, and follow-up duration.

T2DM is characterized by IR and decreased *β*-cell function [3, 4, 24]. Adipocytes and adipose tissue, which produce a host of hormones and cytokines, play a central role in glucose metabolism and lipid metabolism [25]. During feeding, adipocytes synthesize and store triglycerides. During fasting, adipocytes hydrolyze and release triglycerides as free fatty acids (FFAs) and glycerol, which are taken up and oxidized by the skeletal muscle and liver [25, 26]. When IR exists, muscle, liver, adipose, pancreatic *β*-cells, and other tissues contribute to hyperglycemia and hyperlipidemia [27]. During hypertriglyceridemia, triglycerides decrease glucokinase activity and glucose-stimulated insulin secretion in islets [28]. While the islet cells themselves have a weaker antioxidant capacity, hyperglycemia causes islet cells to undergo continuous oxidative stress [29]. Thus, glucose toxicity and lipotoxicity may exert an impact on *β*-cell failure [27]. Simental-Mendía et al. [6] proposed TyG index, which combines fasting triglycerides and fasting blood glucose. In 748 apparently healthy participants, the sensitivity and specificity of TyG index for identifying IR were 84.0% and 45.0%, respectively [6]. Recently, studies have indicated that TyG index is associated with IR and proposed it as a reliable and useful surrogate indicator for identifying IR [7–9]. The association between TyG index and incident diabetes mellitus has been reported in several studies [10–19]. A cohort study with 5354 nondiabetic participants in Korea followed for 4.6 years firstly showed that, compared to participants in the lowest quartile of TyG, participants in the highest quartile of TyG had a higher risk of diabetes (relative risk 4.095; 95% CI 2.701-6.207) after adjusting for age, gender, BMI, WC, SBP, HDL-cholesterol, a family history of diabetes, smoking, alcohol drinking, education, and insulin [15]. Furthermore, they found that the association was stable in both men and women and in obese and nonobese individuals [15]. A rural Chinese cohort had similar results both in men and women [11]. Also, in a cohort of 4820 White Europeans, participants in the highest quartile of TyG had over a fivefold risk of T2DM compared with the lowest quartile, in both men and women, using the multivariate Cox models (HR 5.91, 95% CI 2.26–15.43 for women; HR 5.41, 95% CI 3.26–8.97 for men; *P* for interaction = 0.063) [13]. These conclusions are consistent with our findings (TyG as continuous variable: HR 2.46, 95% CI 1.34-4.53 for women; HR 1.59, 95% CI 1.06-2.37 for men; *P* for interaction = 0.1899). In addition, in a Chinese cohort study of 5706 people with normal body mass index (BMI) (18.5–23.9 kg/m2), after adjusting forwaist circumference and other relevant covariates, the OR and 95% CI for diabetesmellitus associated with TyG were 2.05 (1.23, 3.41) for men and 4.04 (2.76,5.92) for women[10]. This phenomenon mightbe due to the fact that women of all age groups andelderly men havea very high prevalence of central obesity basedon waist circumference and waist-to-height ratio criteria[10]. Similarly,the proportion of womenwith visceral obesity (WC > 90 cm in men and> 80 cm in women)was higher than that of men in this study (11.32% versus 4.73%). In our study, associations between TyG and diabeteswere positive after adjusting for gender and other relevant covariates (HRand 95% CI 1.77 (1.21, 2.57) without visceral obesity; 1.64 (0.76, 3.53) for visceral obesity; P for interaction = 0.848). These findings furtherconfirm our conclusion. Inagreement with our results, Navarro-Gonzalez et al.[13] showed that, despite whether TyG index was assessed as a continuous or categorical variable, a higher TyG index was related to an increased risk ofT2DM after adjustment for other related covariates.Furthermore, Zhang et al.[10]showed thatTyG index was nonlinearly positively related to T2DMrisk in China. However,we found that thedose-response relationship between TyG index and the risk of T2DM was linear. In summary, TyG was positively associated with an increased risk of T2DM after adjusting for confounding factors. Our study has severalstrengths. (1) Compared to previous similar studies, our study had arelatively large sample size. (2) The correlation between TyG index and T2DMwas performed in Japan for the first time. (3) Todecrease the result contingency and elevate the robustness ofthe results, TyG was treated both as a continuous and categoricalvariable. (4) In the subgroup analysis, we used stratified linear regressionmodels and likelihood ration tests to find modifications and interactions andto obtain stable results in different subgroups.

There are some limitations to our study. (1) Our participants were Japanese. Therefore, the universality and extrapolation of the study is weak. (2) We excluded participants who had hepatitis B and C virus, fatty liver, or a heavy drinking habit and participants whose fasting plasma glucose was more than 6.1 mmol/L; therefore, the results of our study cannot be extrapolated for these people. (3) T2DM was not diagnosed by a two-hour oral glucose tolerance test, which may result in an inadequate diagnosis of diabetes. (4) According to BMI values, most of the population in our study was lean. Thus, our findings could be inappropriate for obese and severely obese populations. (5) Raw data were limited. We cannot adjust the family history of diabetes, education, and the intensity and frequency of exercise, which may affect the relationship between TyG index and T2DM. (6) The raw data did not provide information on the types and amounts of foods and beverages (including all types of water) consumed during the 24-hour period prior to examination, which may affect the blood levels of triglycerides and glucose. (7) Data were collected between 2004 and 2015, a difference of almost 10 years between some individuals in this study. However, the association between TyG and T2DM was stable, according to the median of the follow-up day for stratification.

## 5. Conclusion

In conclusion, TyG index was linearly and positively associated with the risk of incident T2DM in the Japanese cohort after adjusting for age, sex, BMI, waist circumference, HDL-cholesterol, total cholesterol, systolic blood pressure, regular exercise, smoking status, and alcohol consumption.

## Figures and Tables

**Table 1:** Baseline characteristics of the study participants sorted by quartiles of TyG.

TyG	Q1	Q2	Q3	Q4	*P* value	*P* value^∗^
Number	3177	3180	3182	3184		
Age (years)	40.11 ± 8.06	43.03 ± 8.73	44.54 ± 9.06	46.20 ± 9.01	<0.001	<0.001
BMI (kg/m^2^)	20.20 ± 2.23	21.00 ± 2.44	21.66 ± 2.53	22.70 ± 2.58	<0.001	<0.001
WC (cm)	70.26 ± 6.68	72.99 ± 7.39	75.22 ± 7.59	79.11 ± 7.46	<0.001	<0.001
HDL-C (mg/dL)	66.09 ± 14.73	62.23 ± 14.74	57.57 ± 14.15	49.33 ± 12.73	<0.001	<0.001
TC (mg/dL)	180.47 ± 29.38	191.61 ± 29.79	199.12 ± 30.91	210.98 ± 33.24	<0.001	<0.001
TG (mg/dL)	30.66 ± 7.71	49.72 ± 6.40	70.61 ± 9.11	130.61 ± 57.75	<0.001	<0.001
FPG (mg/dL)	88.01 ± 6.64	91.00 ± 6.80	93.32 ± 6.69	95.79 ± 6.63	<0.001	<0.001
SBP (mmHg)	107.03 ± 12.49	110.82 ± 13.55	114.16 ± 14.29	117.97 ± 14.24	<0.001	<0.001
DBP (mmHg)	66.11 ± 8.81	68.91 ± 9.58	71.40 ± 9.92	74.25 ± 9.92	<0.001	<0.001
Sex (%)					<0.001	—
Female	2460 (77.43%)	1891 (59.47%)	1381 (43.40%)	816 (25.63%)		
Male	717 (22.57%)	1289 (40.53%)	1801 (56.60%)	2368 (74.37%)		
Regular exercise (%)					0.165	—
None	2612 (82.22%)	2563 (80.60%)	2608 (81.96%)	2632 (82.66%)		
>1×/week	565 (17.78%)	617 (19.40%)	574 (18.04%)	552 (17.34%)		
Smoking status (%)					<0.001	—
Never	2501 (78.72%)	2150 (67.61%)	1788 (56.19%)	1366 (42.90%)		
Past	354 (11.14%)	492 (15.47%)	637 (20.02%)	743 (23.34%)		
Current	322 (10.14%)	538 (16.92%)	757 (23.79%)	1075 (33.76%)		
Alcohol consumption (%)					<0.001	—
None	2779 (87.47%)	2525 (79.40%)	2350 (73.85%)	2063 (64.79%)		
Light	219 (6.89%)	359 (11.29%)	421 (13.23%)	473 (14.86%)		
Moderate	147 (4.63%)	225 (7.08%)	298 (9.37%)	440 (13.82%)		
Heavy	32 (1.01%)	71 (2.23%)	113 (3.55%)	208 (6.53%)		
T2DM (%)					<0.001	—
No	3167 (99.69%)	3156 (99.25%)	3141 (98.71%)	3109 (97.64%)		
Yes	10 (0.31%)	24 (0.75%)	41 (1.29%)	75 (2.36%)		

Values are expressed as mean ± standard deviation or *n* (%). Differences in baseline characteristics were compared with the use of chi-square tests for categorical variables and ANOVA for continuous variables. BMI: body mass index; WC: waist circumference; HDL-C: high-density lipoprotein cholesterol; TC: total cholesterol; TG: triglyceride; FPG: fasting plasma glucose; SBP: systolic blood pressure; DBP: diastolic blood pressure; TyG: triglyceride-glucose index; T2DM: type 2 diabetes mellitus.

**Table 2:** Univariate analysis for diabetes mellitus.

	Statistics	HR (95% CI)	*P* value
Age (years)	43.47 ± 9.01	1.08 (1.07, 1.10)	<0.0001
BMI (kg/m^2^)	21.39 ± 2.62	1.18 (1.12, 1.25)	<0.0001
WC (cm)	74.40 ± 7.97	1.06 (1.04, 1.08)	<0.0001
HDL-C (mg/dL)	58.81 ± 15.43	0.97 (0.96, 0.98)	<0.0001
TC (mg/dL)	195.55 ± 32.81	1.01 (1.00, 1.01)	0.0057
TG (mg/dL)	70.43 ± 47.84	1.01 (1.00, 1.01)	<0.0001
FPG (mg/dL)	92.03 ± 7.28	1.19 (1.16, 1.22)	<0.0001
SBP (mmHg)	112.50 ± 14.24	1.03 (1.02, 1.04)	<0.0001
DBP (mmHg)	70.17 ± 10.03	1.04 (1.03, 1.06)	<0.0001
TyG	7.91 ± 0.60	2.94 (2.28, 3.80)	<0.0001
Sex (%)			
Female	6548 (51.47%)	1.0	
Male	6175 (48.53%)	2.21 (1.56, 3.12)	<0.0001
Regular exercise (%)			
<1/week	10415 (81.86%)	1.0	
≥1/week	2308 (18.14%)	1.08 (0.72, 1.62)	0.7083
Smoking status (%)			
Never	7805 (61.35%)	1.0	
Past	2226 (17.50%)	1.50 (0.96, 2.34)	0.0768
Current	2692 (21.16%)	2.36 (1.66, 3.37)	<0.0001
Alcohol consumption (%)			
None	9717 (76.37%)	1.0	
Light	1472 (11.57%)	0.88 (0.50, 1.54)	0.6503
Moderate	1110 (8.72%)	1.88 (1.17, 3.02)	0.0088
Heavy	424 (3.33%)	4.54 (2.83, 7.29)	<0.0001

BMI: body mass index; WC: waist circumference; HDL-C: high-density lipoprotein cholesterol; TC: total cholesterol; TG: triglyceride; FPG: fasting plasma glucose; SBP: systolic blood pressure; DBP: diastolic blood pressure; TyG: triglyceride-glucose index; T2DM: type 2 diabetes mellitus; CI: confidence interval; HR: hazard ratio.

**Table 3:** Relationship between TyG and incident diabetes mellitus in different models.

Variable	Model 1		Model 2		Model 3	
	HR (95% CI)	*P* value	HR (95% CI)	*P* value	HR (95% CI)	*P* value
TyG	2.94 (2.28, 3.80)	<0.0001	2.26 (1.70, 3.01)	<0.0001	1.79 (1.25, 2.57)	0.0016
TyG						
Q1	Ref		Ref		Ref	
Q2	2.21 (1.06, 4.62)	0.0353	1.73 (0.82, 3.63)	0.1485	1.58 (0.75, 3.33)	0.2311
Q3	3.48 (1.74, 6.95)	0.0004	2.30 (1.13, 4.66)	0.0210	1.85 (0.90, 3.83)	0.0963
Q4	6.29 (3.25, 12.19)	<0.0001	3.59 (1.80, 7.15)	0.0003	2.33 (1.09, 4.96)	0.0282
*P* for trend	<0.0001		<0.0001		0.0224	

Model 1 was not adjusted. Model 2 was adjusted for age and sex. Model 3 was adjusted for age, sex, BMI, WC, HDL-C, TC, SBP, regular exercise, smoking status, and alcohol consumption. TyG: triglyceride-glucose index; CI: confidence interval; HR: hazard ratio; Ref: reference.

**Table 4:** Subgroup analyses of the association between TyG and incident diabetes mellitus.

Subgroup	Participants (*n*)	HR (95% CI)	*P* value	*P* for interaction
Age (years)				
<45	7437	2.26 (1.35, 3.78)	0.0019	0.2057
≥45	5279	1.58 (1.05, 2.38)	0.0299	
Sex				0.1899
Female	6548	2.46 (1.34, 4.53)	0.0039	
Male	6168	1.59 (1.06, 2.37)	0.0237	
Smoking status				0.9218
Never	7803	1.82 (1.11, 3.00)	0.0183	
Past	2225	1.57 (0.79, 3.12)	0.1935	
Current	2688	1.83 (1.11, 3.00)	0.0175	
Alcohol consumption (g/week)				0.1489
0 g/week	3989	2.81 (1.47, 5.38)	0.0017	
>0 g/week	8727	1.73 (1.18, 2.54)	0.0051	
WC (cm)				0.848
<90 in male, <80 in female	11684	1.77 (1.21, 2.57)	0.0029	
≥90 in male, ≥80 in female	1032	1.64 (0.76, 3.53)	0.2071	
BMI (kg/m^2^)				0.2722
<25	11598	1.91 (1.31, 2.80)	0.0009	
≥25	1118	1.27 (0.63, 2.56)	0.5055	
Follow-up duration (day)				0.6791
<1982	6360	1.89 (1.24, 2.90)	0.0032	
≥1982	6356	1.69 (1.08, 2.66)	0.0220	

Notes: adjusted for age, sex, BMI, WC, HDL-C, TC, SBP, regular exercise, smoking status, and alcohol consumption (continuous) except the subgroup variable.

## Data Availability

The data are available at http://www.Datadryad.org/. which allows researchers to freely download the original data.

## Abbreviations

T2DM: Type 2 diabetes mellitus
IR: Insulin resistance
TyG index: Triglyceride-glucose index
BMI: Body mass index
WC: Waist circumference
HDL-C: High-density lipoprotein cholesterol
TC: Total cholesterol
TG: Triglycerides
SBP: Systolic blood pressure
DBP: Diastolic blood pressure.
